# Quality of life in women with female pattern hair loss and the impact of topical minoxidil treatment on quality of life in these patients

**DOI:** 10.3892/etm.2013.1126

**Published:** 2013-05-21

**Authors:** XIAO-SHENG ZHUANG, YOU-YOU ZHENG, JIA-JIA XU, WEI-XIN FAN

**Affiliations:** Department of Dermatology, The First Affiliated Hospital of Nanjing Medical University, Nanjing, Jiangsu 210029, P.R. China

**Keywords:** China, dermatology life quality index, visual analog scale

## Abstract

Female pattern hair loss (FPHL) is the most common hair loss disorder in women and it may impact on the psychological and social activities of patients, thereby reducing their quality of life (QoL). Topical minoxidil has been shown to be effective and safe in the treatment of patients with FPHL. The aim of this study was to assess the QoL of patients with FPHL and investigate whether topical minoxidil solution treatment improves the QoL of these patients. In this study, we enrolled 125 female patients aged 16–72 years to answer visual analog scale (VAS) and dermatology life quality index (DLQI) questionnaires. Of these patients, 31 were recruited for the follow-up study after 12 months of treatment with 2% minoxidil. Each index and the change in QoL prior to and following treatment were statistically analyzed. There was identified to be a correlation between clinical severity and the values of the indices in all patients. There was a statistically significant difference between the VAS and DLQI scores prior to and following treatment with 2% minoxidil. A comparison between the good responders (n=23) and the poor responders (n=8) revealed no significant difference in the improvement of VAS and DLQI scores. The QoL of the patients was severely impaired by FPHL. The DLQI and VAS used in this study were validated as useful indices for the evaluation of QoL due to their high reliability, sensitivity and simplicity. This evaluation is recommended for the management of FPHL treatment. The results of the study demonstrated that topical minoxidil improved the QoL of the patients.

## Introduction

Female pattern hair loss (FPHL), characterized by a diffused reduction in hair thickness, is the most common cause of hair loss in women and affects >50% of women at the age of 80 (1). Furthermore, an epidemiological survey in northern China suggested that the incidence was 6.0% in females, lower than that in Western countries (2). However, due to the large population in China, the number of women with FPHL in China is higher. Although it is a mild dermatological disorder, psychologists and dermatologists have observed that even clinically imperceptible hair loss is capable of damaging the quality of life (QoL) of patients due to the loss of self-image and diminished self-esteem (3,4). Women with hair loss have reported experiencing adverse psychosocial reactions, including irritability, anger, anxiety and depression, due to the significant sexual and social functions of the appearance of their hair (5–8).

Treatment options are currently limited and a substantial length of time is required to reach a satisfactory outcome. FDA-approved minoxidil topical solution is an effective and safe treatment for female androgenetic alopecia which acts by promoting hair follicle cycling and prolonging aging (9,10). The treatment needs to be continued for at least 12 months prior to performing an accurate efficacy evaluation (11). However, few studies have examined the impact of topical minoxidil treatment on the QoL of female patients. This study was designed to assess the QoL in patients with FPHL (n=125) and determine the effectiveness of topical minoxidil treatment in improving the QoL of these patients (n=31).

## Patients and methods

### Patients and study design

We recruited 125 FPHL patients to answer visual analog scale (VAS) (12) and dermatology life quality index (DLQI) questionnaires (13). Of these, 31 patients were recruited for the follow-up study in which they were treated with topical 2% minoxidil solution (1 ml applied twice daily) for 12 months and then completed the same surveys. The general characteristics of the patients are described in Table I. This study was approved by the Nanjing Medical University Institutional Review Board. Written consent was obtained from all patients prior to enrollment.

### Study assessments

Two indices were used to evaluate the improvement of patients receiving treatment for 12 months. Each patient was assessed as one of four grades, namely: ‘significant improvement’, ‘improvement’, ‘no change’ and ‘worsening’ A ‘significant improvement’ was defined as an increase in the Ludwig score by two, while ‘improvement’ was defined as an increase in the Ludwig score by one. A reduction of the score by one or two was considered ‘worsening’. Patients assessed as having a ‘significant improvement’ or ‘improvement’ were defined as ‘good responders’ while those deemed to have ‘no change’ or to be ‘worsening’ were defined as ‘poor responders’. The improved value of each QoL index was compared with the baseline.

### DLQI

We modified certain items in the index to ensure it was appropriate for alopecia patients (Table II). One of the endpoints of the study was the mean DLQI score for the first-visit patients. The other major endpoint was the mean improvement in the DLQI score from the baseline to 12 months later. Each question has four alternative responses with corresponding scores of 0, 1, 2 and 3, respectively. The DLQI was calculated by summing the scores of all questions, with total scores ranging from 0–30, where a lower score indicates a lower QoL.

### VAS

Subjective assessment (the patient’s perception of hair loss severity) of the 125 first-visit patients and a comparison between the assessment results prior to and following treatment were conducted using a VAS (8,12,14–16) in which the patient’s evaluation was scored from 0 (completely dissatisfied) to 100 (completely satisfied). The VAS is a simple tool for measuring the satisfaction of the patients regarding the state of their hair loss and the effect of the treatment.

### Statistical analyses

The Kruskal-Wallis Test was used to analyze the correlation between clinical characteristics (age, duration and severity) and the QoL of the patients. Pearson correlation coefficients determined whether the opinion of the patient regarding their hair loss correlated with the DLQI (the correlation between the indices). The indices prior to and following the treatment were compared using the Student’s paired t-test. Comparison of the improved value of each QoL index of the good responders and poor responders from the baseline was performed using the Student’s unpaired t-test. The data are presented as the means ± SD.

## Results

### Correlation between clinical characteristics and the QoL of first-visit patients

**Figure 1.**
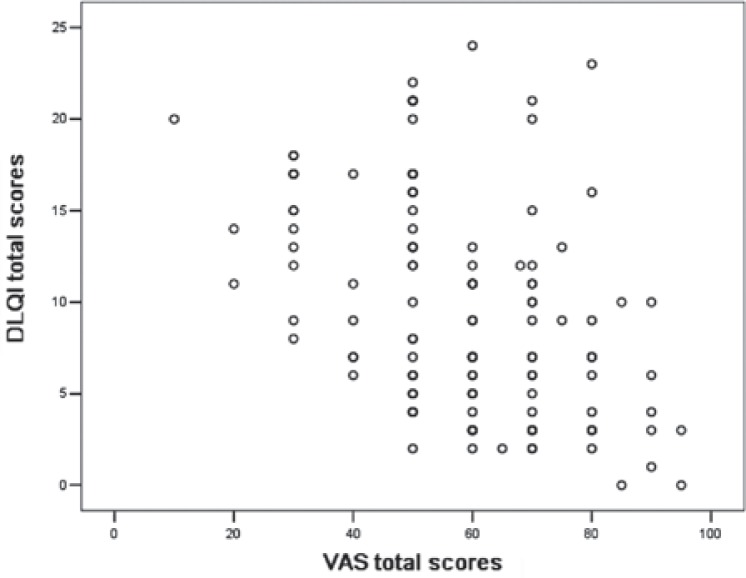
Correlation between DLQI and VAS. P<0.000, r=−0.441, Pearson analysis. DLQI, dermatology life quality index; VAS, visual analog scale.

All patients were classified using the Ludwig criteria. Analysis of the correlation between clinical characteristics (age, duration and severity) and VAS and DLQI values was performed on the 125 first-visit patients. The scores for the VAS and DLQI indices were 57.78±18.06 and 9.62±5.92, respectively. There was no correlation between age, duration and the two indices. However, the group with the greatest severity also had the highest DLQI total score (P<0.05, Kruskal-Wallis Test) and the lowest score on the VAS scale (P<0.05, Kruskal-Wallis Test; Table III). The DLQI and VAS scales correlated markedly with a statistically significant negative correlation in all first-visit patients (P<0.000, r=−0.441, Pearson correlation; Fig. 1).

### Comparison of DLQI and VAS scores prior to and following the administration of topical minoxidil

**Figure 2.**
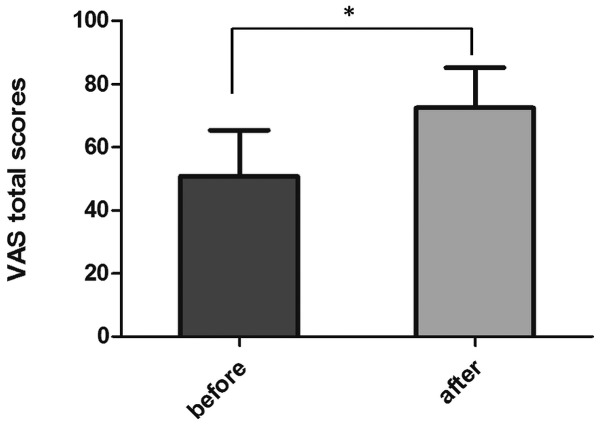
Comparison of visual analog scale (VAS) scores prior to and following the administration of topical minoxidil. ^*^P<0.01, n=31, Student’s paired t-test. Data presented are the mean±SD.

**Figure 3.**
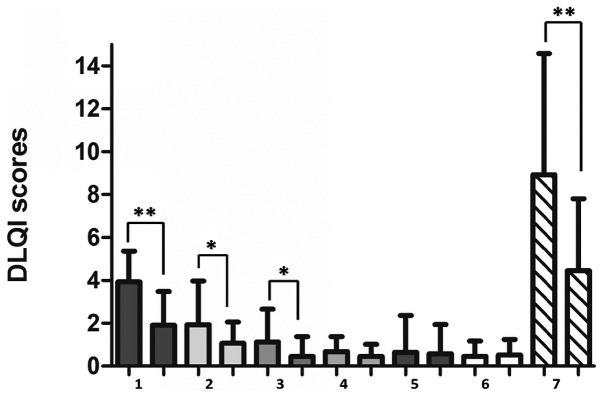
Comparison of dermatology life quality index (DLQI) scores prior to and following the administration of topical minoxidil: 1, symptoms and feelings; 2, daily activities; 3, leisure; 4, work and school; 5, personal relationships; 6, treatment and 7, total score. ^*^P<0.05, ^**^P<0.01, n=31, Student’s paired t-test. Data presented are the means ± SD.

We analyzed the responses to the VAS and DLQI questionnaires from 31 patients prior to and following treatment. The scores for the VAS indices prior to and following the administration of minoxidil were 50.81±14.61 and 72.52±12.79 (P<0.000, n=31, Student’s paired t-test), respectively (Fig. 2). The total scores for the DLQI indices were 8.94±5.65 and 4.45±3.36, prior to and following treatment, respectively. The difference between DLQI scores was statistically significant (P<0.01, n=31; Student’s paired t-test). With regard to specific life events, there were significant differences in the scores for ‘symptoms and feelings’ (P<0.01, lane 1), ‘daily activities’ (P<0.01, lane 2) and ‘leisure’ (P<0.05, lane 3; Fig. 3).

### Comparison between good and poor responders

**Figure 4.**
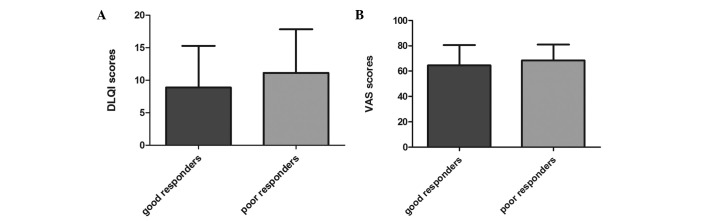
Comparison between good responders and poor responders. (A) Improved value of dermatology life quality index (DLQI) total score (P=0.403). (B) Improved value of visual analog scale (VAS) (P=0.53). Good responders in 23 cases; poor responders in 8 cases. There were no statistically significant differences between the VAS and DLQI total scores (Student’s unpaired t-test). Data are presented as the means ± SD.

The results of the evaluation performed by clinicians were: ‘significant improvement’ in four cases; ‘improvement’ in 19 cases; ‘no change’ in five cases and ‘worsening’ in three cases. In total, 23 cases were assessed as good responders and eight as poor responders. The improved values for VAS from the baseline were 64.57±15.95 in the good responders and 68.5±12.46 in the poor responders. The improved values for the DLQI total scores from the baseline were 8.87±6.41 in the good responders and 11.13±6.71 in the poor responders (Fig. 4). No significant differences were observed between the scores of the good responders and poor responders assessed by Student’s unpaired t-test.

## Discussion

Multiple clinical trials have confirmed that topical minoxidil ameliorates hair loss in FPHL patients. Our study demonstrated that such treatment also improves the QoL of patients. Yamazaki *et al* (12) verified that DLQI and VAS are two highly reliable and sensitive indices for assessing the improvement of QoL in male patients with androgenic alopecia following oral finasteride treatment. We used their study as a reference when conducting an assessment of the QoL in female Chinese patients.

To explore the QoL of FPHL patients, we investigated 125 first-visit patients with a total DLQI score of 9.62±5.92, which was close to 10 [>10 indicates a very severe impact (17)]. This value is higher than that in a previous study (12), which reported a value of 5.74±6.14 in male patients; however, the value is similar to the score for decubitus, while exceeding the scores for atopic dermatitis and psoriasis (18). Collective evidence has suggested that FPHL impairs the QoL of female patients to the same extent as certain lifelong skin disorders such as psoriasis. Studies by Cash *et al* (5) and van der Donk *et al* (19) reported that those seeking treatment for FPHL experienced social anxiety disorder more severely, which is consistent with our observation that FPHL has a greater impact on the QoL and psychology in female patients than in male patients. Women are more likely to suffer from mental illness as they pay more attention to their self-image in society (12).

To identify the correlations between the clinical characteristics of the patient and the severity of hair loss, we classified all patients using the Ludwig criteria and observed that there was a significant correlation between the clinical severity and the VAS and DLQI indices while there were no correlations between the other factors such as age and disease duration. The more severe the clinical rating of hair loss, the greater the impact on the DLQI and VAS scores. To a certain extent, these results revealed that the clinical severity of hair loss may affect the QoL of a patient. However a previous study (20) revealed that younger male patients with a longer duration of hair loss had a reduced QoL, which contradicts our results. This may be due to gender differences in psychological needs and clinical features. Moreover, our finding demonstrated that DLQI negatively correlated with VAS, implying that the subjective rating for the severity of hair loss markedly synchronized with the QoL in female patients. This suggests that dermatologists should consider the subjective satisfaction and objective rating in their estimation of clinical severity.

To further understand the impact of topical minoxidil on the QoL of female patients, we compared the scores of the VAS and DLQI indices prior to and following treatment. The VAS scores were 50.81±14.61 and 72.52±12.79 prior to and following treatment, respectively, while the total scores for the DLQI indices were 8.94±5.65 and 4.45±3.36, respectively. A significant difference in the evaluations demonstrated that topical minoxidil may improve the QoL of female patients, particularly in areas of their lives such as ‘symptoms and feelings’, ‘daily activities’ and ‘leisure’. In a previous study (21), women developed certain coping mechanisms following the acceptance of hair loss, such as avoiding negative emotions from their surroundings by reducing their outdoor activities or by wearing hats or wigs to prevent discomfort. Therefore it is unsurprising that effective clinical treatments are able to improve the scores of corresponding items in the questionnaire. No statistical difference in the scores for ‘treatment’ item of the questionnaire implied that the use of topical minoxidil did not disturb the life of the patient, as it was easy to use and did not clutter the patient’s home, which should be mentioned in clinical use.

We classified the 31 patients into two groups according to their response to the treatment. There were no statistically significant differences between the DLQI and VAS scores of the good responders and poor responders, revealing that subjective satisfaction and QoL do not always correspond with objective evaluation in female patients following treatment. This is consistent with the results from the aforementioned study of male patients (12). Subjective satisfaction is considered to be determined by various psychological factors and a potential variable is the motivation for treatment. For instance, patients with an intrinsic reason (for example, improvement of self-image) may experience greater satisfaction than others with external requirements (wanting to please others or to increase job opportunities) (22). The initial expectation of the treatment is another factor, due to the fact that certain patients may have expectations that go beyond medical limitations and are therefore likely to lead to disappointment. Moreover, the length of the treatment prior to any apparent improvement (increased terminal hair count) may not necessarily result in cosmetic changes that satisfy certain patients (23).

Overall, dermatologists should be alert to the following potentially significant factors: sufficient time should be spent on consultation and at the first visit, patients should take a few minutes to complete a simple questionnaire regarding their motivation and expectations of the treatment in order to promote communication between the doctor and patient. Clinicians should answer any questions the patient may have to ease their concerns regarding hair loss and to correct any impractical expectations, particularly regarding the improvement of hair quantity and time taken for hair growth. DLQI and VAS are simple and reliable surveys for understanding the patient’s feelings and satisfaction prior to and following treatment. However, due to the ‘placebo effect’, certain patients may be satisfied with the treatment even if they are poor responders, implying that dermatologists should take the psychological requirement of the patient into account throughout the therapeutic process.

In conclusion, evidence has suggested that patients with hair loss experience a significantly impaired QoL, however, few studies have focused on women with FPHL. Our findings observe that a reduced QoL is a substantial part of the disease in patients with FPHL and topical minoxidil solution may improve the QoL. The current study presents instructive and meaningful information to dermatologists and hair experts with regard to the benefits of integrating the psychological factors of a patient into their clinical treatment. However, further studies concerning the impact of FPHL treatment on self-esteem, self-image and QoL are required in the future.

## Figures and Tables

**Table I.** Patient characteristics (n=125).

Characteristic	Value
Age (years)	32.210±10.351
Disease duration (months)	31.770±34.446
Ludwig score (cases)	
I	57
II	46
III	22

Data for age and disease duration are the mean ± SD.

**Table II.** DLQI and VAS questionnaire.

No.	Question
1	Over the last week, how have you been affected by alopecia? Have you felt burning, pain, itching, irritation or oils on your scalp?
2	Over the last week, how embarrassed, frustrated or self conscious have you been because of your alopecia?
3	Over the last week, how much has your alopecia interfered with your shopping or other outdoor activities?
4	Over the last week, how much has your alopecia influenced your hair style? Do you need to wear a hat, wig or special hair type to cover the thinner area?
5	Over the last week, how much has your alopecia affected any social or leisure activities?
6	Over the last week, how much has your alopecia made it difficult for you to do any sport or hobbies?
7	Over the last week, has your alopecia prevented you from working or studying?
8	Over the last week, how much has your alopecia created problems with your partner or any of your close friends or relatives?
9	Over the last week, how much has your alopecia caused any sexual difficulties?
10	Over the last week, how much of a problem has the treatment for your alopecia been, for example by making your home messy or taking up time?
11	Assess your alopecia condition yourself from score 0–100 (VAS).

1 and 2, symptoms and feelings; 3 and 4, daily activities; 5 and 6, leisure; 7, work and school; 8 and 9, personal relationships; 10, treatment. DLQI, dermatology life quality index; VAS, visual analog scale.

**Table III.** Correlation between single variables and QoL.

Variable	DLQI	VAS
Age (years)		
<30	10.300±5.873	55.870±19.462
30–50	9.590±5.996	56.480±18.722
>50	9.250±4.979	56.250±10.607
P-value	0.797	0.946
Disease duration (months)		
<12	10.520±5.999	59.130±16.832
12–24	9.480±6.583	55.000±17.705
>24	9.860±5.842	56.390±18.652
P-value	0.672	0.764
Severity (Ludwig score)		
I	7.950±5.280	64.790±17.050
II	9.820±6.130	56.050±15.300
III	12.530±5.830	49.000±15.610
P-value	<0.050	<0.001

Kruskal-Wallis test. Data presented are the mean ± SD. QoL, quality of life; DLQI, dermatology life quality index; VAS, visual analog scale.
